# Causal relationship between antihypertensive drugs and Hashimoto’s thyroiditis: a drug-target Mendelian randomization study

**DOI:** 10.3389/fendo.2024.1419346

**Published:** 2024-10-07

**Authors:** Bing Cui, Aqin Chen, Chengcheng Xu, Chaoming Mao, Yuehua Chen

**Affiliations:** ^1^ Department of Blood Transfusion, Affiliated Hospital of Jiangsu University, Zhenjiang, Jiangsu, ;China; ^2^ Department of Nuclear Medicine, Affiliated Hospital of Jiangsu University, Zhenjiang, Jiangsu, ;China

**Keywords:** antihypertensive drugs, Hashimoto’s thyroiditis, Mendelian randomization, drug-Target, SNP

## Abstract

**Introduction and objectives:**

Recent studies have indicated a potential association of hypertension with Hashimoto’s thyroiditis (HT) and other autoimmune diseases, yet the impact of antihypertensive drugs on HT risk is not well understood.

**Methods:**

We employed a drug-target Mendelian randomization approach to investigate the prolonged impact of 9 classes of antihypertensive medications on HT susceptibility in European and Asian populations. Genetic variants close to or within genes associated with the drug targets and systolic blood pressure (SBP) were utilized to mimic the effects of antihypertensive medications. We focused on drugs linked to a lower risk of coronary artery disease for our main analysis. We gathered genetic data on SBP and HT risk from comprehensive genome-wide association studies available for European and Asian groups. For a supplementary analysis, we used expression quantitative trait loci (eQTLs) related to drug target genes as proxies.

**Results:**

Our analysis revealed that the use of calcium channel blockers (CCBs) is linked to a reduced risk of HT in both European (OR [95% CI]: 0.96 [0.95 to 0.98] per 1 mmHg decrease in SBP; p = 3.51×10^-5^) and Asian populations (OR [95% CI]: 0.28 [0.12, 0.66]; p = 3.54×10^-3^). Moreover, genetically mimicking the use of loop diuretics (OR [95% CI]: 0.94 [0.91, 0.97]; p = 3.57×10^-5^) and thiazide diuretics (0.98 [0.96, 0.99]; p = 3.83×10^-3^) showed a significant association with a decreased risk of HT only in European population. These outcomes were confirmed when eQTLs were employed to represent the effects of antihypertensive medications.

**Conclusion:**

The study suggests that CCBs and diuretics could potentially reduce the risk of HT in different populations. Additional research is needed to assess the feasibility of repurposing antihypertensive medications for the prevention of HT.

## 1 Introduction

Hashimoto’s thyroiditis (HT), also known as chronic lymphocytic or autoimmune thyroiditis, manifests with an enlarged thyroid gland, lymphocytic infiltration into the thyroid parenchyma, and antibodies targeting thyroid-specific antigens. Together with Graves’ disease, it forms a subset of autoimmune thyroid disorders, whose prevalence has notably increased in recent years (1–3). Currently, HT is the leading cause of hypothyroidism (4). Additionally, individuals with HT have a higher risk of developing cardiovascular diseases and malignant tumors (5, 6). Although the exact mechanisms underlying HT remain largely unclear, genetic factors, environmental triggers, and epigenetic modifications influence its pathogenesis (7).

The interaction between hypertension and autoimmune thyroiditis has attracted clinical interest due to observed overlaps in their epidemiological and pathological features (8–10) Thyroid dysfunction resulting from HT is associated with various cardiovascular risk factors, including hypertension, indicating potential shared pathways influenced by genetic, environmental, or pharmacological factors (11, 12). Antihypertensive drugs, primary in managing high blood pressure, are believed to impact thyroid function, particularly beta-blockers, known for their effects on thyroid hormone metabolism and symptoms of thyroid diseases (13, 14). HT’s underlying pathobiology, involving pro-inflammatory and pro-angiogenic pathways, and disruptions in immune-endocrine interactions, strongly links to cardiovascular disease risk. Dysfunctions in autophagy and mitophagy processes, impacting apoptosis and angiogenesis, may contribute to this, consistent with our previous research (15, 16). The effects of antihypertensive drugs are not limited to blood pressure regulation, they may also influence the development of HT by modulating certain pathological processes, either directly or indirectly. For instance, calcium channel blockers, due to their anti-inflammatory properties, may reduce systemic inflammation and lessen the immune system’s attack on thyroid tissue (17, 18). Diuretics, on the other hand, may impact HT by regulating electrolyte balance and influencing immune system function (19, 20). Therefore, the appropriate selection of antihypertensive drugs not only helps manage cardiovascular risk but may also play a key role in the prevention and management of HT.

A connection between inflammation and hypertension exists (21), with studies showing higher inflammatory markers in plasma of essential hypertension patients compared to normotensive individuals (22–24). Hypertension contributes significantly to vascular remodeling, triggering an inflammatory response in arterial walls (25, 26). This process elevates circulating inflammatory markers, angiotensinogen, and angiotensin II levels, further increased by thyroid hormone, corticosteroids, and estrogen in endothelial cells (27). Inflammatory responses in blood vessels are crucial for vascular remodeling, potentially leading to increased blood pressure (28). Consequently, ongoing systemic inflammation in HT may influence hypertension likelihood. Notably, the impact of antihypertensive medication on HT remains minimally explored in epidemiological studies, with traditional pharmaco-epidemiological research vulnerable to biases compromising validity and reliability.

Mendelian randomization (MR) evaluates causal relationships between modifiable exposures or risk factors and clinically meaningful outcomes using genetic variations (29). The resultant estimate is consistent even in cases of reverse causation and unmeasured confounding if the instrumental variable conditions are met (30). MR reduces bias for confounding variables in observational research, being more practical than randomized controlled trials. Drug-target MR predicts treatment outcomes and side effects before clinical trials. It uses genetic variants as proxies for drug target effects, enabling investigation of protein functions and drug target alterations. This technique assessed long-term effects of nine antihypertensive medications on HT in European and Asian populations to determine their causal relationships with the disease.

## 2 Methods

### 2.1 Study design

**Figure 1:**
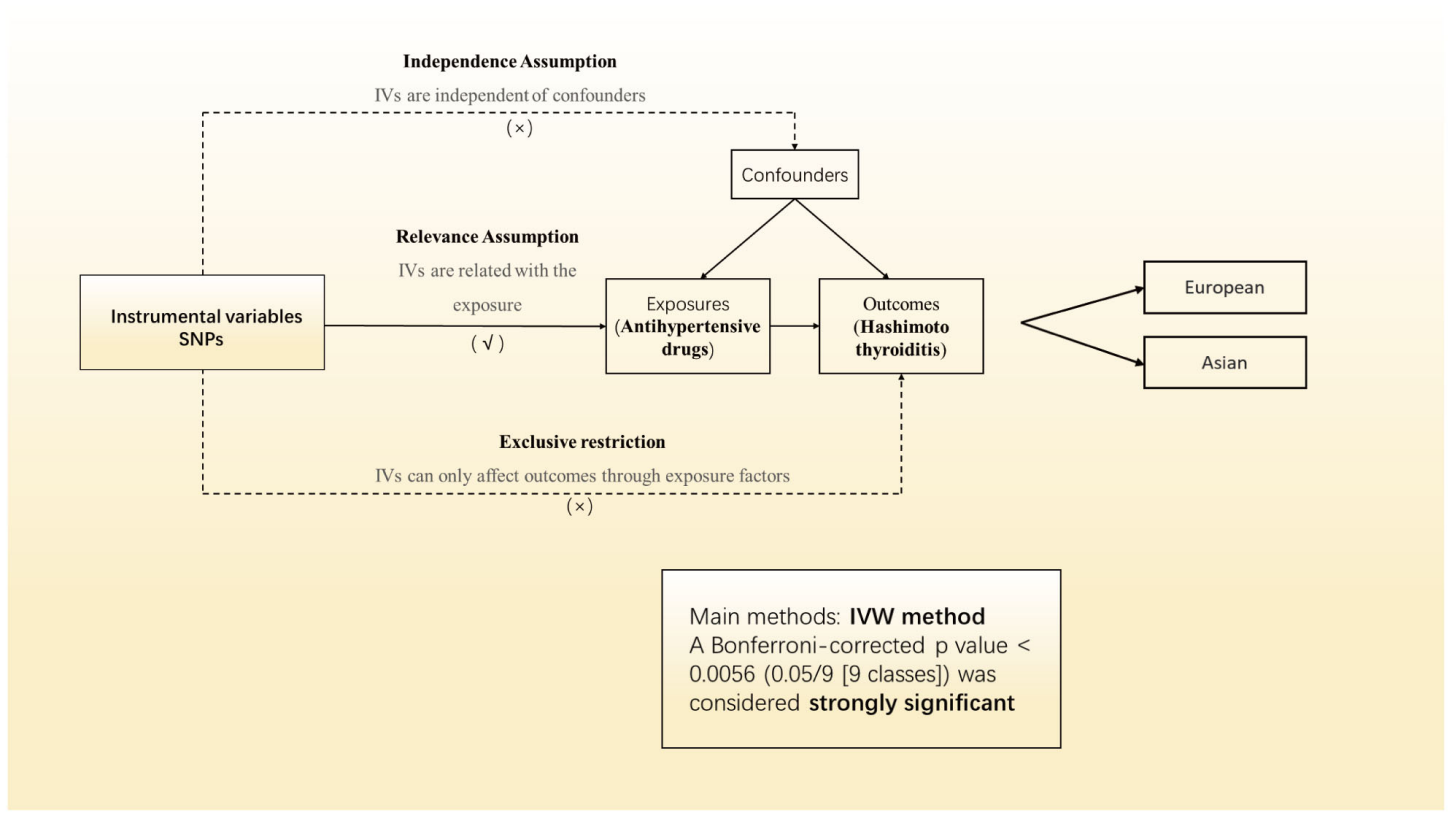
Study design of the MR study of the associations between genetically proxied antihypertensive drugs and the risk of Hashimoto thyroiditis.

**Figure 1** illustrates the workflow of our current drug-target Mendelian Randomization (MR) investigation. Initially, we consulted the British National Formulary to compile a comprehensive list of antihypertensive medication categories, then utilized the DrugBank database to pinpoint the specific target genes for each category. Following this, we employed genetic markers to represent 8 categories of antihypertensive medications. These genetic markers were derived from two separate Genome-Wide Association Studies (GWAS) focusing on systolic blood pressure (SBP) from a European consortium. Next, we conducted an MR analysis to explore the relationship between genetically inferred antihypertensive medication use and the incidence of coronary artery disease (CAD), thus evaluating the efficacy of our genetic markers across different cohort. Antihypertensive medications that showed no correlation with CAD risk were omitted from further analysis. The concluding phase entailed examining the influence of genetically inferred antihypertensive medication categories on the risk of HT in both European and Asian populations, utilizing the validated genetic markers. Institutional Review Board (IRB) Approval (or Waiver) Statement: This study utilized summary statistics data exclusively, without any individual-level data involvement, hence no ethical approval was necessary.

### 2.2 Identification of target genes for antihypertensive drug classes

We sourced information on 9 antihypertensive drug categories from the British National Formulary. These categories include adrenergic neuron blockers, alpha-adrenoceptor blockers, ACE inhibitors (ACEi), angiotensin II receptor blockers (ARBs), beta-blockers (BBs), calcium channel blockers (CCBs), loop diuretics, potassium-sparing diuretics and aldosterone antagonists, and thiazide diuretics and related compounds (31). To identify the target genes for the active components within each antihypertensive class, we utilized the DrugBank database (DrugBank). The compilation of these antihypertensive classes alongside their respective target genes is detailed in **Supplementary Table S1**.

### 2.3 Selection of genetic instruments for proxy antihypertensive drug

To mimic the effects of antihypertensive drugs genetically, we focused on selecting single nucleotide polymorphisms (SNPs) situated near or within the drug target gene regions, which are linked to systolic blood pressure (SBP) variations. For individuals of European descent, these genetic markers were obtained through a combined analysis of GWAS data on SBP from the International Consortium of Blood Pressure (ICBP) and the UK Biobank, encompassing 757,601 individuals of European background (32). Similarly, genetic instruments for Asians were extracted from GWASs of SBP in the biobank Japan project. Following established criteria, we chose genome-wide significant SNPs (p < 5 × 10^-8^) located within a ±100 kb radius of the target genes for each drug class, ensuring these SNPs had low linkage disequilibrium (r^2^ < 0.1 within 100 kb) with each other (33). This selection strategy aimed to enhance the explained variance for each antihypertensive drug class through the genetic markers and to strengthen the instruments’ effectiveness. Specifically, it was deemed acceptable to utilize SNPs with an r^2^ < 0.1 as proxies to represent the various classes of antihypertensive medications.

We assessed the F-statistics for each single nucleotide polymorphism (SNP) related to genetically mimicked antihypertensive medications. SNPs exhibiting F-statistics less than 10 were removed to mitigate the risk of weak instrument bias, as F-statistics equal to or above 10 suggest a robust instrument. In instances where an SNP from the genetic proxy was absent in the outcome genome-wide association study (GWAS), we sought a substitute SNP that was in strong linkage disequilibrium (r^2^ > 0.80) via LDlink (LDlink). Should a fitting proxy SNP not be found, that SNP was then excluded from analysis. To ensure consistency between the exposure and outcome data, SNP effects were harmonized, aligning them with the same effect allele across both datasets. Throughout this harmonization effort, SNPs that were ambiguous or palindromic (with a minor allele frequency > 0.42) were systematically omitted.

### 2.4 Outcome data

In this study, coronary artery disease (CAD) served as the control outcome, reflecting the pivotal role of antihypertensive medications in decreasing CAD morbidity. For Europeans, summary-level CAD data were derived from a combined analysis of genome-wide association studies (GWAS) conducted by the CARDIoGRAMplusC4D consortium and the UK Biobank, encompassing 122,733 CAD cases and 424,528 controls (34).

The primary focus of the research was HT. Summary-level HT data were obtained from a GWAS involving 395,640 individuals of European descent, which included 15,654 HT cases and 379,986 controls. HT was classified according to the International Classification of Diseases, Ninth Revision (ICD-9) and Tenth Revision (ICD-10), with codes 245.2 and E06.3 respectively for each revision (35). The GWAS utilized the BOLT-LMM method for analysis, and the resulting association statistics, initially presented on a linear scale, were converted into log odds ratios using a standardized transformation process.

In the case of Asian population, summary-level HT data came from the same study of 173,193 individuals, which recorded 537 HT cases and 172,656 controls. The original research of GWAS was a multi-institutional, hospital-based registry that compiled DNA, serum, and clinical data, genotyped with Illumina HumanOmniExpressExome BeadChip or a combination of the Illumina HumanOmniExpress and HumanExome BeadChip. Diagnosis of HT cases was conducted by attending physicians (35).

### 2.5 Statistical analysis

Initially, we assessed the validity of SNPs for genetically mimicking 9 antihypertensive drug classes by setting these classes as exposures and using CAD as a reference outcome. Upon data harmonization, the inverse-variance weighted (IVW) approach was utilized to determine the impact of these genetically mimicked antihypertensive drug classes on CAD. Any class of genetically mimicked antihypertensive drugs not showing an inverse relationship with CAD risk at a nominally significant level (p ≥ 0.05) was removed to affirm the legitimacy of the genetically mimicked drugs.

Subsequently, we applied the IVW method to explore the association between validated genetically mimicked antihypertensive drugs and HT risk among the two ethnic cohorts. The IVW method, when adopting a multiplicative random-effects model, offers a more accurate estimate and confidence interval (CI) compared to the fixed-effect IVW approach in cases of heterogeneity. The fixed-effect IVW method is preferred if no heterogeneity is present; otherwise, the multiplicative random-effects IVW model is employed. To evaluate heterogeneity, the Cochran Q test and I^2^ statistics were conducted (36). The primary findings were expressed as odds ratios (ORs) for disease occurrence per 1 mmHg decrease in SBP attributed to the antihypertensive drug class. The Bonferroni correction method was applied to adjust for multiple comparisons, setting a p-value threshold of < 0.0056 (0.05/9, considering 9 classes of antihypertensive drugs and one cancer outcome) to denote “strongly significant” Results falling between a p-value of ≥ 0.0056 and < 0.05 were classified as “suggestively significant”.

In the final stage of our analysis, we employed cis-expression quantitative trait loci (cis-eQTLs) as genetic surrogates to measure exposure to each antihypertensive drug class in European populations, using data from previous studies (31). These studies identified SNPs that regulate the expression of drug target genes, based on data from the Genotype-Tissue Expression (GTEx) consortium. The selected SNPs demonstrated an impact on systolic blood pressure (SBP) according to summary statistics from the UK Biobank. Consequently, we excluded trans-eQTLs and SNPs with linkage disequilibrium (r^2^ > 0.1) based on the 1000 Genomes Project Phase 3 European reference panel to maintain specificity. To verify the approach, we assessed the relationship between eQTLs that mimic antihypertensive drug exposure and SBP, utilizing summary statistics from the International Consortium for Blood Pressure (ICBP) and the UK Biobank. The inverse-variance weighted (IVW) method was used to estimate the effect size, representing the change in SBP per standard deviation decrease in the gene expression level. Moreover, we applied the IVW method to investigate the causal links between the expression levels of drug target genes and the risk of HT in European populations. The outcomes were articulated as odds ratios (ORs) for HT per 1 mmHg reduction in SBP, which is influenced by the expression of antihypertensive drug target genes. This approach aimed to elucidate the potential genetic basis of antihypertensive drug efficacy in reducing HT risk through blood pressure modulation.

To assess the potential for horizontal pleiotropy, we also performed the MR-Egger regression and the MR Pleiotropy Residual Sum and Outlier (MR-PRESSO) test (37, 38). The average pleiotropic effect of IVs is indicated by the intercept term in the MR-Egger regression (38). To test for heterogeneities, we employed MR-egger regression and Cochran’s Q statistic. In addition, the robustness and consistency of the findings were evaluated using the leave-one-out method.

The packages “TwosampleMR” and “MRPRESSO” in R version 4.2.2 were used for all of the analyses.

## 3 Results

### 3.1 Genetic instrument selection

**Figure 2:**
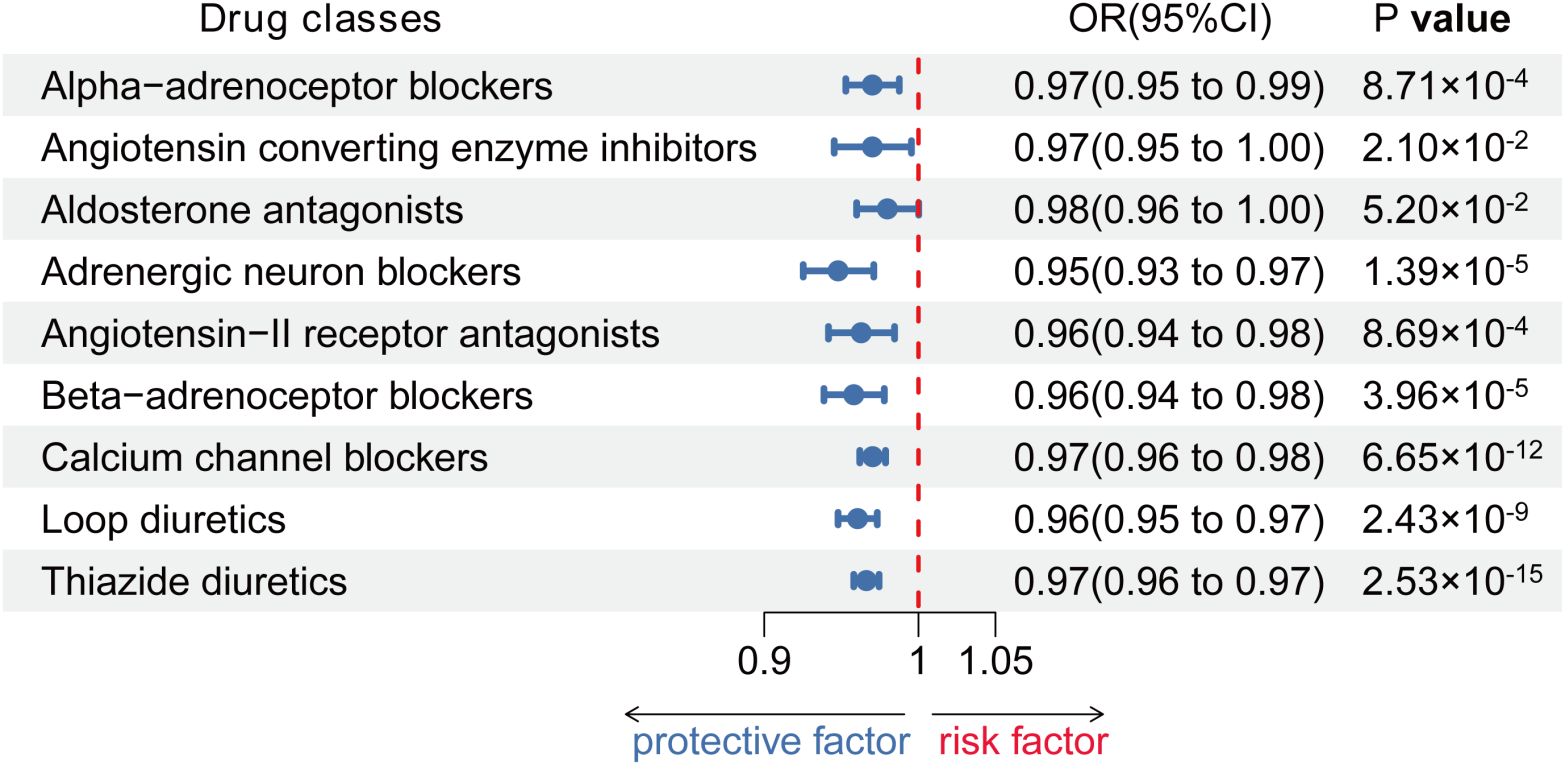
Associations between genetic proxies for 9 classes of antihypertensive drugs and the risk of CAD.

For our study, we identified a range of 7 to 155 SNPs associated with systolic blood pressure (SBP) to serve as genetic proxies for 9 different classes of antihypertensive drugs in European and Asian populations, detailed in **Supplementary Tables S2**, **S3**. The F-statistics for all chosen SNPs exceeded 10, which is indicative of their robustness and the reliability of these genetic instruments (as documented in **Supplementary Tables S2**, **S3**). Among the 9 classes of antihypertensive drugs modeled genetically, 8 showed a significant association with the risk of coronary artery disease (CAD) in the European cohort. However, aldosterone antagonists did not display this association. Despite this, they were retained for comprehensive analysis in the study, as illustrated in **Figure 2**. Although the observed effect sizes are relatively small, their clinical significance should not be overlooked. In the context of a multifactorial disease like Hashimoto’s thyroiditis (HT), even modest reductions in risk can have a substantial impact on public health, especially in high-risk populations. Moreover, the cumulative effect of reducing multiple small risks may significantly lower the overall incidence of HT.

### 3.2 Causal effect of genetically proxied antihypertensive drugs and HT

**Figure 3:**
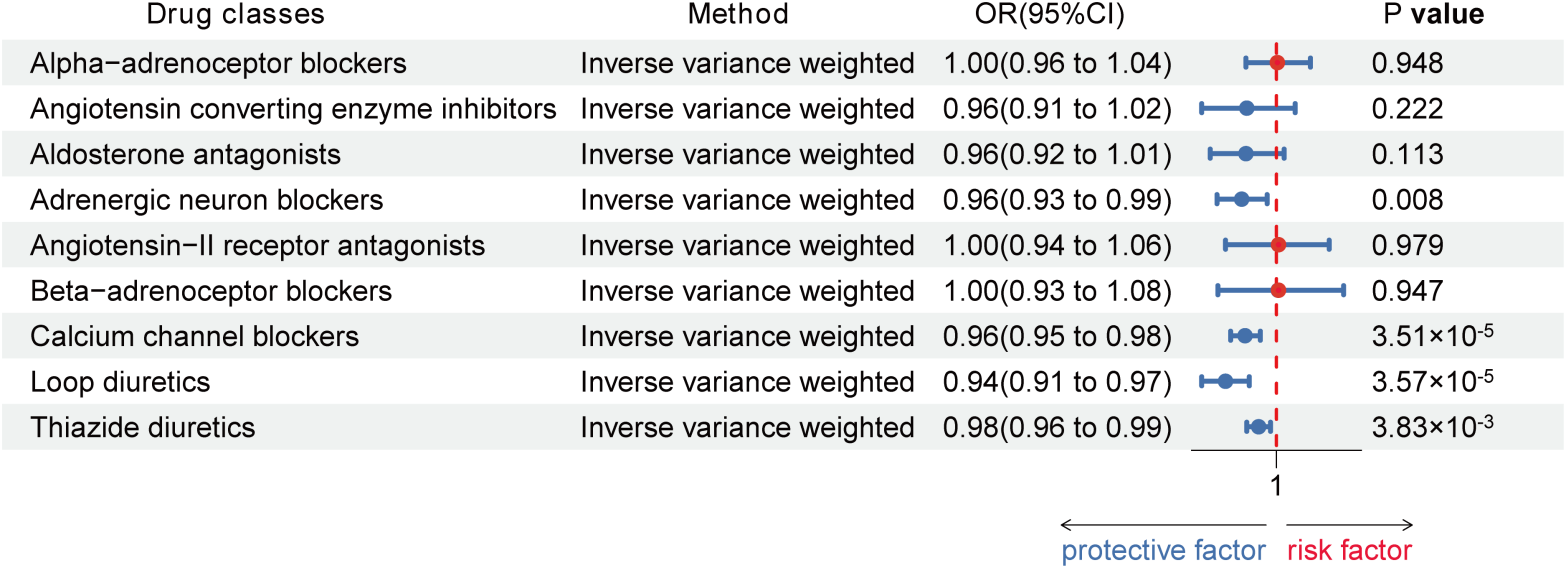
Associations between genetic proxies for 9 classes of antihypertensive drugs and the risk of Hashimoto thyroiditis in European.

**Figure 4:**
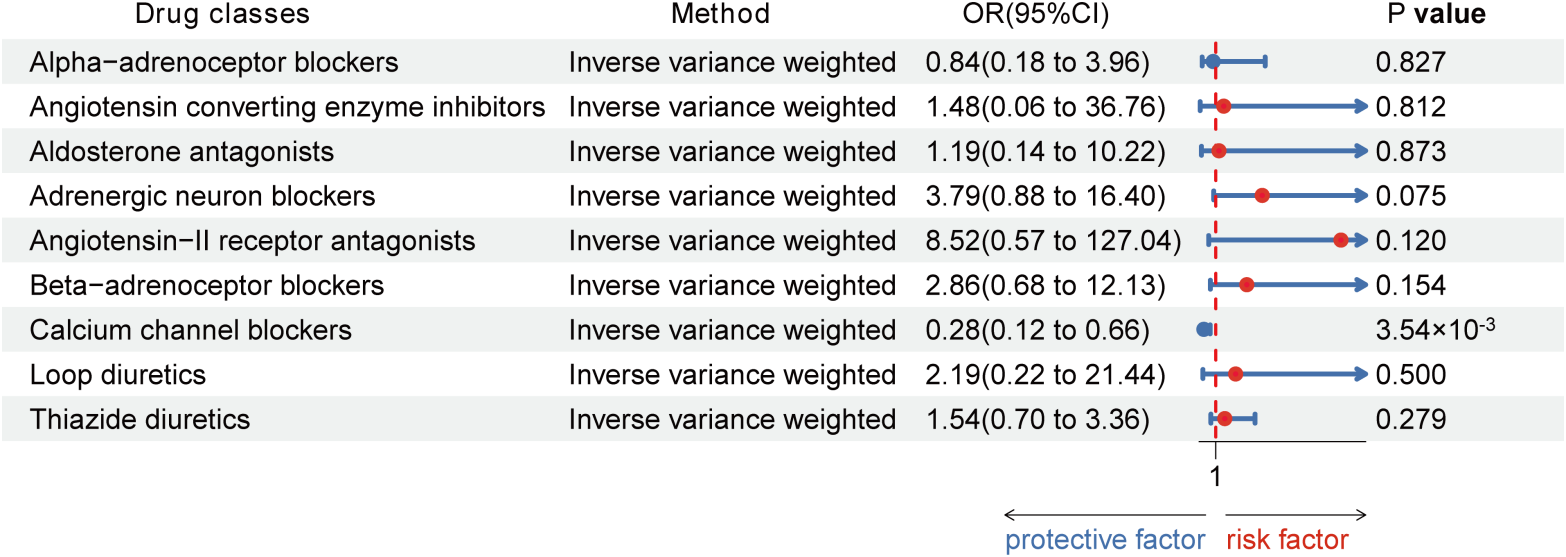
Associations between genetic proxies for 9 classes of antihypertensive drugs and the risk of Hashimoto thyroiditis in Asian.

Using the Inverse-Variance Weighted (IVW) method, we discovered robust evidence linking genetically proxied CCBs with a reduced risk of HT in both European (Odds ratio [OR] [95% Confidence Interval]: 0.96 [0.95 to 0.98] per 1 mmHg decrease in SBP; p = 3.51×10^-5^) and Asian (OR [95% CI]: 0.28 [0.12, 0.66] per 1 mmHg reduction in SBP; p = 3.54×10^-3^). Moreover, genetically proxied loop diuretics (OR [95% CI]: 0.94 [0.91, 0.97] per 1 mmHg decrease in SBP; p = 3.57×10^-5^) and thiazide diuretics (0.98 [0.96, 0.99] per 1 mmHg decrease in SBP; p = 3.83×10^-3^) showed a significant association with a decreased risk of HT in Europeans, but showed no association in Asians (**Table 1**; **Figures 3**, **4**).

**Table 1:** Primary results for MR of Genetic proxies for 9 antihypertensive drugs with Hashimoto thyroiditis.

Drugs	Beta	SE	P value	OR	95% CI
European					
Alpha-adrenoceptor blockers	0.0013	0.0195	0.9483	1.0013	0.964-1.040
Angiotensin converting enzyme inhibitors	-0.0354	0.0290	0.2223	0.9652	0.912-1.022
Aldosterone antagonists	-0.0365	0.0231	0.1137	0.9642	0.922-1.009
Adrenergic neuron blockers	-0.0414	0.0157	0.0082	0.9594	0.930-0.989
Angiotensin-II receptor antagonists	-0.0008	0.0311	0.9790	0.9992	0.940-1.062
Beta-adrenoceptor blockers	0.0025	0.0377	0.9474	1.0025	0.931-1.079
Calcium channel blockers	-0.0373	0.0090	**3.51E-05**	0.9634	0.946-0.981
Loop diuretics	-0.0622	0.0151	**3.57E-05**	0.9397	0.912-0.968
Thiazide diuretics	-0.0213	0.0074	**3.83E-03**	0.9790	0.965-0.993
Asian					
Alpha-adrenoceptor blockers	-0.1733	0.7911	0.8266	0.8409	0.178-3.964
Angiotensin converting enzyme inhibitors	0.3898	1.6401	0.8121	1.4767	0.059-36.756
Aldosterone antagonists	0.1746	1.0966	0.8735	1.1908	0.139-10.217
Adrenergic neuron blockers	1.3319	0.7477	0.0749	3.7881	0.875-16.400
Angiotensin-II receptor antagonists	2.1420	1.3788	0.1203	8.5169	0.571-127.038
Beta-adrenoceptor blockers	1.0513	0.7369	0.1536	2.8615	0.675-12.129
Calcium channel blockers	-1.2636	0.4332	**3.54E-03**	0.2826	0.121-0.661
Loop diuretics	0.7848	1.1636	0.5000	2.1920	0.224-21.444
Thiazide diuretics	0.4308	0.3982	0.2793	1.5385	0.705-3.358

These findings remained consistent across various robustness checks, including the weighted median, and MR-PRESSO methods, as detailed in **Supplementary Table S1**. The MR-PRESSO global test found no evidence of horizontal pleiotropy. However, heterogeneity in the effect estimates of genetic proxies for alpha-adrenoceptor blockers on HT risk was observed in European population (p for Cochran Q value = 43.751; P value = 0.016) and aldosterone antagonists in Asian population (p for Cochran Q value = 47.022; P value = 0.007), necessitating the use of IVW with multiplicative random effects to mitigate this issue (**Table 2** and **Supplementary Table S4**). Sources of heterogeneity may include differences in the frequency of genetic variants across populations, diversity in drug metabolism pathways, and the interaction of environmental factors. These differences can lead to variations in drug mechanisms across populations, affecting the interpretability of study results. Future research could incorporate stratified analyses and introduce interaction terms to identify and quantify potential factors contributing to heterogeneity. These analytical methods would help us better understand the sources of these differences and increase our confidence in the results.

**Table 2:** Heterogeneity and pleiotropy results.

Drugs	Heterogeneity		Pleiotropy		
	Q	P value	Egger_intercept	SE	P value
European					
Alpha-adrenoceptor blockers	43.751	0.016	-0.001	0.013	0.912
Angiotensin converting enzyme inhibitors	3.841	0.698	0.011	0.022	0.631
Aldosterone antagonists	5.680	0.772	-0.014	0.018	0.478
Adrenergic neuron blockers	24.337	0.442	-0.035	0.025	0.284
Angiotensin-II receptor antagonists	9.895	0.272	0.031	0.028	0.299
Beta-adrenoceptor blockers	15.626	0.058	-0.026	0.024	0.308
Calcium channel blockers	16.551	0.083	0.003	0.007	0.685
Loop diuretics	31.196	0.221	-0.012	0.015	0.418
Thiazide diuretics	28.461	0.497	0.012	0.006	0.044
Asian					
Alpha-adrenoceptor blockers	10.471	0.988	0.016	0.047	0.731
Angiotensin converting enzyme inhibitors	5.115	0.529	-0.006	0.111	0.958
Aldosterone antagonists	47.022	0.007	0.006	0.070	0.932
Adrenergic neuron blockers	14.636	0.931	-0.010	0.050	0.835
Angiotensin-II receptor antagonists	6.290	0.615	0.006	0.084	0.941
Beta-adrenoceptor blockers	24.573	0.487	0.004	0.043	0.931
Calcium channel blockers	58.819	0.991	-0.025	0.028	0.372
Loop diuretics	14.123	0.441	-0.051	0.058	0.394
Thiazide diuretics	77.794	0.871	0.009	0.023	0.699

We employed the MR-PRESSO and MR-Egger methods to detect potential pleiotropy and other sources of bias. Notably, the MR-Egger regression intercept for thiazide diuretics in the European population showed slight significance, suggesting the possibility of some pleiotropy. However, this finding did not substantially alter the robustness of the overall analysis. This result strengthens our confidence in the validity of the results obtained through the IVW method. Furthermore, the global test of MR-PRESSO did not reveal significant horizontal pleiotropy, further supporting the robustness of the IVW findings. MR-PRESSO also confirmed most of the primary causal relationships identified by the IVW method, ensuring the reliability of the study conclusions in the face of potential pleiotropy (**Table 2**).

### 3.3 Causal effect of expressions of target genes of antihypertensive drugs and HT

Upon refining the cis-expression quantitative trait loci (cis-eQTLs) from previous research by clumping (r^2^ < 0.1), we identified eQTLs for the 9 classes of antihypertensive drugs, detailed in **Supplementary Table S5**. Consistently, reduced expression levels of the target genes for these antihypertensive drug classes correlated with lower systolic blood pressure (SBP) across the board (all p < 0.001).

Furthermore, our analysis revealed that expression of target genes for CCBs was linked to lower risks of HT in European population (Beta: -0.69 [−1.35,−0.03] per 1 mmHg decrease in SBP; p = 0.041) while expression level of target genes for beta−adrenoceptor blockers was linked to increased risks of HT in European population (Beta: 0.87 [0.02, 1.73] per 1 mmHg decrease in SBP; p = 0.045) (**Supplementary Figure 1**). However, expression levels of target genes for CCBs and beta−adrenoceptor blockers were not observed to be associated with risk of HT in Asian population (**Supplementary Figure 2**).

## 4 Discussion

This MR investigation provides substantial evidence for the beneficial role of genetically inferred CCBs and thiazides and related diuretics in reducing the risk of HT across different study, with results corroborated by MR analyses and eQTL analyses linking the expression of target genes for classes of antihypertensive drugs with HT. The findings suggest that CCBs, loop diuretics, and thiazide diuretics, due to their potentially favorable safety profiles as antihypertensive treatments, could be considered for HT prevention strategies. However, the study does not provide definitive evidence linking genetic proxies for primary antihypertensive treatments, such as ACE inhibitors (ACEi) and angiotensin receptor blockers (ARBs), or other medications, with the decreasing risk of HT.

Our Mendelian randomization study provides groundbreaking insights into the potential role of antihypertensive medications, specifically CCBs and two types of diuretics, in reducing the risk of HT. This finding is particularly significant given the current scarcity of observational studies examining the relationship between antihypertensive drugs and HT. Our research not only fills this gap but also opens new avenues for therapeutic interventions.

**Figure 5:**
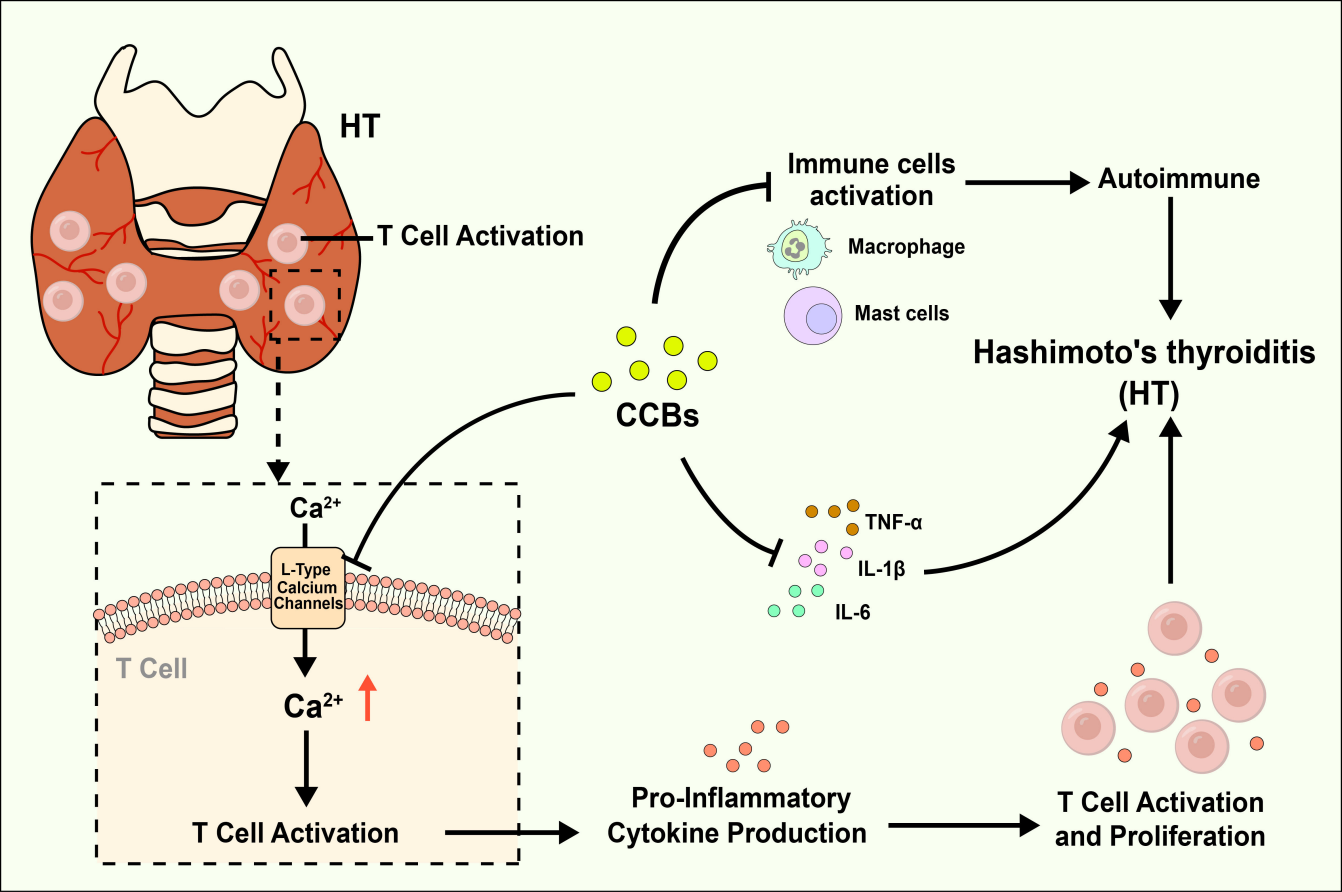
Calcium channel blockers (CCBs) might reduce the risk of Hashimoto thyroiditis.

The absence of prior observational research into the link between these antihypertensive classes and HT risk might be attributed to a focus on more established risk factors and treatments. However, the implications of our findings suggest that cardiovascular pharmacotherapy could play an unexpected role in managing an endocrinologic disorder characterized by its complexity and multifactorial etiology, including inflammatory, hormonal, genetic, and environmental components. The mechanisms through which CCBs and diuretics may exert protective effects against HT are yet to be fully understood. However, several plausible hypotheses can be proposed. For one, CCBs are known to possess anti-inflammatory properties (39, 40), which could help mitigate the chronic inflammation associated with HT. The mechanism by which CCBs might confer protection against Hashimoto’s thyroiditis could be multifaceted. Calcium ions play a pivotal role in the activation and proliferation of T cells, which are central to the autoimmune response observed in Hashimoto’s (41, 42). By inhibiting L-type calcium channels, these drugs could theoretically reduce T cell activation and thus, dampen the autoimmune attack on the thyroid gland. Furthermore, CCBs have been shown to exert anti-inflammatory effects, which may further contribute to their protective role (43, 44). In addition to T-cell pathways, CCBs may exert potential protective effects in HT through other anti-inflammatory and immunomodulatory mechanisms. Studies suggest that CCBs might reduce autoimmune attacks on the thyroid by decreasing the activation of various immune cells, including mast cells and macrophages. Furthermore, CCBs may inhibit the calcineurin/NFAT pathway and reduce the production of pro-inflammatory cytokines, thereby further attenuating inflammatory responses. These immunomodulatory effects may not be directly related to their antihypertensive action, but by reducing inflammation and immune activation, CCBs could potentially lower the incidence or progression of HT (45–48), Mechanism diagram is shown in **Figure 5**. The protective effect of loop and thiazide diuretics might be more indirect. These drugs are known to alter electrolyte balance, which could, in turn, affect immune system function. For example, changes in potassium and sodium balance can influence the acid-base status and cellular function, potentially modulating immune responses (49, 50). Additionally, thiazide diuretics have been reported to possess anti-inflammatory properties, which might also play a role in reducing autoimmune activity (51).

Although the above hypotheses are not yet supported by experimental evidence, these preliminary findings highlight the need for further research to validate the observed associations and elucidate the mechanisms by which CCBs and diuretics may reduce the risk of Hashimoto’s thyroiditis. Future investigations should include larger, more diverse cohorts to strengthen the evidence base. Importantly, clinical trials are crucial to assess the efficacy, safety, and optimal dosing of these antihypertensive drugs when repurposed as treatments for HT.

Furthermore, while research exploring the association between antihypertensive medications and HT remains limited, there is emerging evidence to suggest that certain antihypertensive drugs may be linked to other autoimmune disorders, such as rheumatoid arthritis and systemic lupus erythematosus. Notably, HT has been identified as a potential risk factor for these conditions or often co-occurs with them. This interconnection underscores the complexity of autoimmune diseases and highlights the potential systemic impact of antihypertensive medications beyond their primary cardiovascular effects.

The observation that antihypertensive drugs might have implications for a range of autoimmune conditions, including rheumatoid arthritis (52–54), and systemic lupus erythematosus (55–57) suggests a broader, perhaps systemic mechanism of action. The association between antihypertensive medications and reduced risk of autoimmune diseases is not entirely novel. Previous studies have hinted at the potential immunomodulatory effects of these drugs, particularly in the context of rheumatoid arthritis and systemic lupus erythematosus (58, 59). Our findings extend this body of evidence, suggesting that such immunomodulatory effects may also apply to Hashimoto’s thyroiditis.

Although the protective effect of CCBs has shown consistency across European and Asian populations, suggesting a potentially universal mechanism, these drugs may act as modifiable risk factors for HT, possibly independent of genetic background. Given the chronic nature of HT and its significant impact on quality of life, identifying modifiable risk factors is crucial for developing prevention strategies. However, we also observed that the effects of other drug categories, such as loop diuretics and thiazide diuretics, were not replicated in the Asian population. This discrepancy may arise from differences in genetic background, environmental factors, and the diversity of drug metabolism pathways between populations (60–62). For example, genetic polymorphisms related to drug metabolism in the Asian population may result in different metabolic responses to these drugs compared to the European population, affecting their efficacy. Additionally, factors such as dietary habits and environmental exposures may contribute to varying drug responses among different populations. These differences remind us that even when drug treatment strategies show population-wide consistency, population-specific factors should be considered when applying them across different groups. Moreover, the possibility that HT may serve as a risk factor or a comorbid condition for other autoimmune diseases further emphasizes the need for a comprehensive approach in the research and treatment of women’s health. Understanding the potential dual benefits of antihypertensive medications could lead to more holistic treatment strategies, addressing both cardiovascular and immune system health simultaneously.

There are several benefits to our research. Firstly, the study employed genetic variants that simulate the effects of antihypertensive medications to assess drug impacts on HT via MR, addressing challenges such as reverse causation and confounding factors inherent in observational studies, and circumventing the extensive time and resources required for randomized controlled trials (RCTs). Secondly, it capitalized on GWAS data from the most comprehensive genetic studies available, bolstering the statistical robustness and credibility of its findings. Thirdly, the investigation meticulously selected genetic variants within drug target genes linked to systolic blood pressure as surrogates for antihypertensive treatments, incorporating a positive control analysis to confirm the appropriateness of these genetic proxies. Lastly, a series of sensitivity analyses were executed to ensure the findings’ reliability and consistency.

Despite these promising findings, the study has several limitations. The use of summary-level data may obscure individual-level variability and introduce bias in the presence of population heterogeneity. Additionally, our approach of selecting SNPs within a ±100 kb region around the target genes may limit the comprehensiveness of the analysis. Moreover, our study did not consider certain factors, such as environmental and inflammatory influences, which may affect the drug targets and potentially lead to adverse effects and long-term health consequences. The relatively small sample size in the Asian database may have reduced the statistical power, especially in assessing the effects of certain drugs, which could impact the robustness and generalizability of our findings.

Future research should not only integrate individual-level data, longer follow-up periods, and larger, more diverse populations to strengthen the evidence base, but also pay particular attention to high-risk groups or subgroups with specific biomarker characteristics, as these populations may benefit more from targeted interventions. Furthermore, it is necessary to explore the mechanisms behind the differential responses to different drug categories across various genetic backgrounds, as well as the interactions between environmental and genetic factors that may influence drug efficacy. To effectively translate these findings into clinical practice, future studies should validate the efficacy of these drugs through RCTs in diverse populations. Additionally, using real-world data to assess long-term outcomes and safety will provide critical support for clinical practice.

## 5 Conclusion

In conclusion, our study highlights the potential association between the use of CCBs, loop diuretics, and thiazide diuretics and a reduced risk of Hashimoto’s thyroiditis. These findings suggest a potential immunomodulatory role for these antihypertensive medications, offering new insights into the prevention and management of autoimmune thyroid disorders. Further research is warranted to explore the mechanisms underlying these associations and to evaluate the clinical implications of our findings. By uncovering these associations, we not only contribute to the understanding of HT pathophysiology but also pave the way for innovative treatment strategies.

## Data Availability

The original contributions presented in the study are included in the article/**Supplementary Material**. Further inquiries can be directed to the corresponding author.

## Supplementary material

Supplementary Figure 1 Associations of expressions of antihypertensive drug classes target genes with Hashimoto thyroiditis in European.

Supplementary Figure 2 Associations of expressions of antihypertensive drug classes target genes with Hashimoto thyroiditis in Asian.
