# Supplementary figures and images for: Causal relationship between antihypertensive drugs and Hashimoto’s thyroiditis: a drug-target Mendelian randomization study
Source: Front Endocrinol (Lausanne). 2024 Oct 7;15:1419346. doi: 10.3389/fendo.2024.1419346 (PMC11491371; doi:10.3389/fendo.2024.1419346)

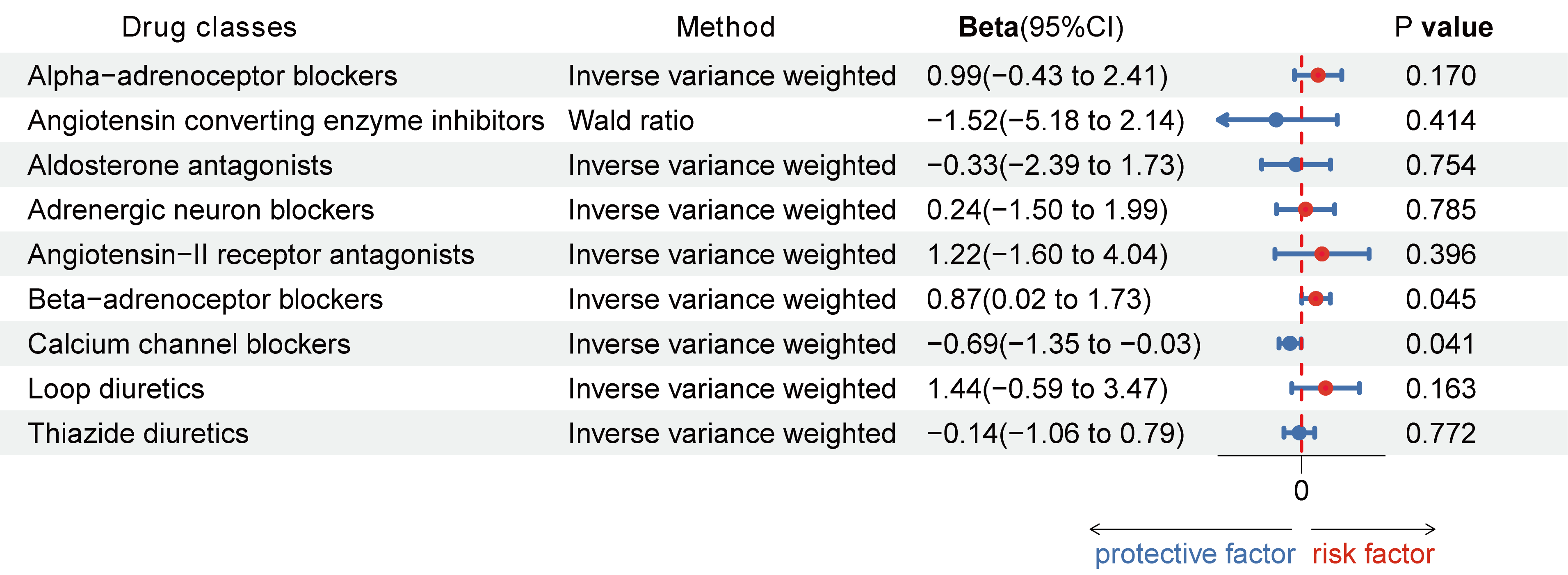

Supplement: Supplementary Figure 1 — Associations of expressions of antihypertensive drug classes target genes with Hashimoto thyroiditis in European. [file Image1.tif]

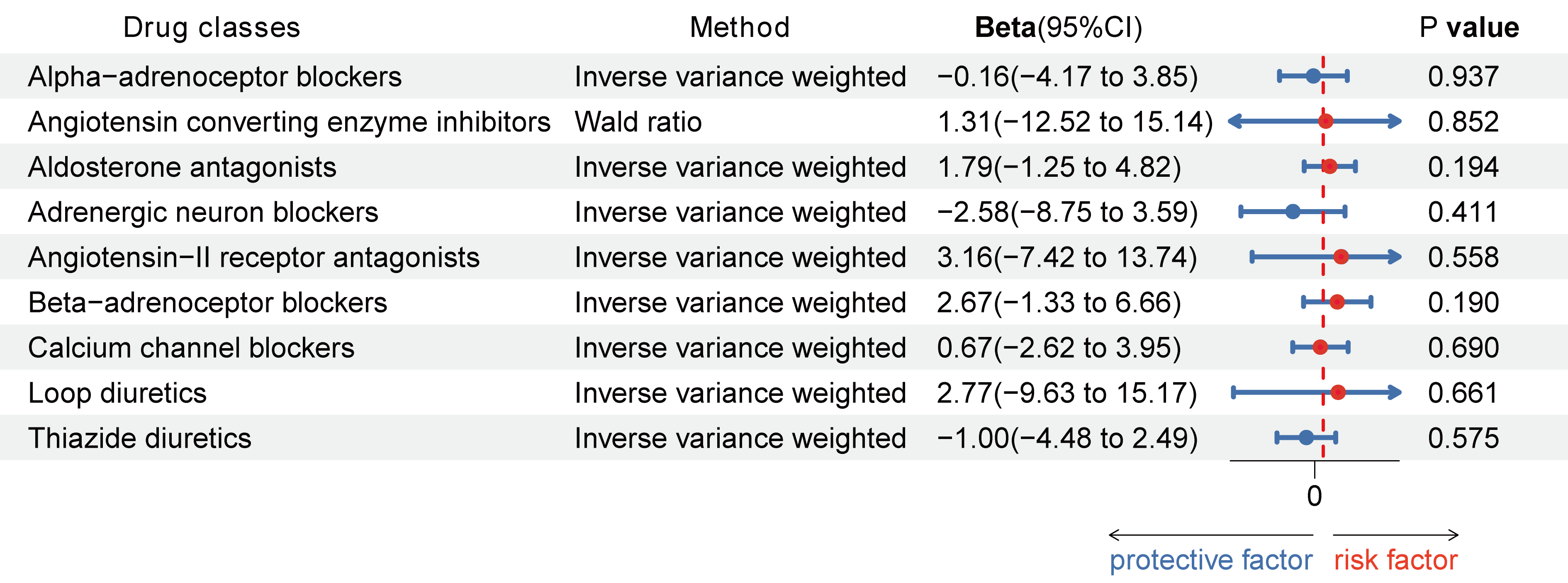

Supplement: Supplementary Figure 2 — Associations of expressions of antihypertensive drug classes target genes with Hashimoto thyroiditis in Asian. [file Image2.tif]
